# The tracking of dietary intakes of children and adolescents in Sweden over six years: the European Youth Heart Study

**DOI:** 10.1186/1479-5868-6-91

**Published:** 2009-12-11

**Authors:** Emma Patterson, Julia Wärnberg, John Kearney, Michael Sjöström

**Affiliations:** 1 Unit for Preventive Nutrition, Department of Biosciences and Nutrition, Karolinska Institutet, 14157 Huddinge, Sweden; 2 School of Biological Sciences, Dublin Institute of Technology, Dublin 2, Ireland; 3 Department of Preventive Medicine and Public Health, University of Navarra, 31080 Pamplona, Spain

## Abstract

**Background:**

The stability of dietary habits through various life-stages is not well understood. A better understanding of the tracking of diet over time could have implications for health promotion as well as for the planning of nutritional epidemiology studies. We examined the stability of dietary intakes of children and adolescents over six years.

**Methods:**

As part of the European Youth Heart Study, in 1998-9, a 24-h dietary recall was performed on over one thousand 9- and 15-year-olds in Sweden. In 2004-5, 40% returned to the follow-up study. These 452 subjects (273 15- and 179 21-year-olds) were assigned to age- and gender-specific tertiles of intakes of food groups, energy, selected nutrients and energy density (low, mid and high) at each time point. The agreement between the classification of subjects into tertiles at both time points was examined using Cohen's weighted κ and other stability coefficients. We included a dropout analysis and considered the effect that energy mis-reporting might have on our results.

**Results:**

Fair tracking was seen between childhood and adolescence for the milk, fil and yoghurt food group (κ = 0.30), and between adolescence and young adulthood for fruit (κ = 0.24). Slight tracking was observed for most other food groups and fair to slight tracking for all nutrients studied. Only membership of the high milk, fil and yoghurt tertile could be predicted from membership at baseline, in children. Excluding potential energy mis-reporters did not affect the results.

**Conclusions:**

Despite the long time between data collections, and the method of dietary data collection used, evidence for slight tracking was observed for most food groups and nutrients over these six years.

## Background

Many established biological cardiovascular disease risk factors are known to track, or remain relatively consistent, with age; for example, high cholesterol, low physical fitness and high body mass index [1,2]. Despite the recognised association between diet and cardiovascular disease risk factors, less is known about the conservation of dietary habits in children and adolescents [3-6]. Assumptions about the formation and stability of dietary habits during childhood and adolescence are often used as the basis for costly and labour-intense behavioural interventions [7]. The evidence for this stability in relation to dietary habits in young people is limited, and most studies report only poor to moderate tracking.

Tracking is defined as "stability over time" [8]. It can be thought of as the position a person holds relative to his or her peers at one time point, to that held at another time point. A better understanding of this phenomenon is important for several reasons. For the promotion of healthy eating it is necessary to know if there are certain life-stages at which young people are more amenable to messages and when changes effected may persist for a long time. For the planning of longitudinal epidemiological studies it is important to know how often dietary assessments should be made, as if, for example, periods of stability are identified, fewer assessments may be necessary [9].

One of the barriers to studying tracking is that cohort or long-term studies are required, and these tend to be less common than cross-sectional studies. The European Youth Heart Study [10] was designed to study the development and progression of cardiovascular risk factors in otherwise healthy children and adolescents as they transition to adolescence and young adulthood, respectively. It therefore provided a valuable opportunity to examine dietary habits and their behaviour over time. We examined the intakes of food groups, energy and selected nutrients in a sample of children and adolescents, followed-up after six years, and here we report the tracking of those intakes.

## Methods

### Study design

Data collection for the Swedish part of the European Youth Heart Study (EYHS) was conducted in 1998-99 (EYHS I) and 2004-5 (EYHS II). Over two thousand (n = 2 313) children (from grade 3, mean age 9 years) and adolescents (grade 9, mean age 15 years) were sampled from classes selected from 42 schools in southern Stockholm and Örebro. Of these, 1 137 students consented to participate in EYHS I, and six years later 459 agreed to take part in EYHS II. Permission was obtained from the local ethical committees (Huddinge University Hospital, permit no. 474/98; Örebro City Council, no. 690/98). Data collection was performed in schools in EYHS I, and on site at Karolinska Institutet (Stockholm) and Örebro University for EYHS II. Written consent was provided by a parent or legal guardian and verbal consent was provided by all 9- and 15-year-old subjects; written consent was provided by the 21-year-old subjects in EYHS II. A more detailed description of the study and the sampling procedure [11] has been published. Complete dietary data was available at both time points for 452 subjects: 273 younger subjects (9 years old, followed up when 15), and 179 older subjects (15 years old, followed up when 21).

### Dropout analysis

Subjects from EYHS I who participated in EYHS II ("participants") did not differ from those who did not ("dropouts") in relation to physical activity, physical fitness or anthropometric indices at EYHS I [12]. A separate dropout analysis focusing on dietary intake found no major discrepancies between the diets of participants and dropouts (Patterson et al, unpublished results). Significant differences were seen in the percent consumers of one food group (out of 19) and in one diet-related frequency question (of 11), after Bonferroni correction for multiple comparisons. Percent energy from carbohydrate was marginally higher in the participants than dropouts (p = 0.04) but neither other macronutrients, energy intake, energy density nor inadequate energy reporting differed between the two groups (p > 0.05).

### Dietary data collection

The subjects completed a single 24-h recall, conducted by a trained interviewer. In EYHS I, the 9-year-old children kept a 1-d qualitative food diary with the assistance of their parents on the day before the interview, to aid as a prompt in case of difficulties with the recall. Otherwise, the protocol remained unchanged between EYHS I and II. A food atlas with pictures of common foods in various portion sizes was available during the interview, along with standard household units to help estimate quantities accurately. Data from the recall was analysed using the dietary analysis software Stor MATs (Rudans Lättdata, Västerås, Sweden); version 4.02 based on the Swedish Food Administration's http://www.slv.se nutritional database 99.1 (EYHS I), and version 4.06 with database 02.1 (EYHS II). Each food consumed was allocated to a food group based on nutritional or dietary similarities and intakes are presented at food group level. Most food groups are self-explanatory, with some clarifications: the "milk, fil, yoghurt" group includes fil, a soured-milk product similar to yoghurt, common in Nordic countries; "sweetened drinks" includes sugar-sweetened carbonated or cordial-based drinks; "fruit juices" refers to concentrated or fresh juices; the "other sweet foods" group includes desserts, ice-cream, sweet soups, jams and added sugar; "cereals" refer to breakfast cereals (including porridge); fried potatoes are included in the "chips, crisps" group. Someone who reported eating any amount of a food group was considered a consumer of that food group. Energy density was calculated as the total energy from food (kJ) consumed divided by the total weight of the food (g). Food was defined as solid food and liquids consumed as food (e.g. soups, yoghurt). All beverages, both energy-containing and non-energy-containing were excluded from this calculation. Energy density was used as a marker of overall dietary quality [13].

Age- and gender-specific tertiles of food group intakes, energy, selected nutrients and dietary energy density were calculated at each time point and subjects were deemed to have low, medium or high intakes, relative to their peers.

### Energy reporting

In order to identify subjects who may have mis-reported dietary intakes to a significant degree, we used the method proposed by Goldberg and colleagues [14] and calculated a 95% confidence interval around predicted intakes required for weight maintenance. Subjects with energy intakes above or below these upper and lower values were deemed "inadequate" reporters, and those within the cut-offs, "adequate" reporters. Physical activity level values and other data required for the calculation of Goldberg cut-offs were taken from Black [15]. In our study, 9-year-olds were defined as adequate reporters if they had a ratio of reported energy to predicted energy of between 0.97 and 2.91 (girls) or between 0.98 and 3.08 (boys); 15-year-olds had cut-offs of 0.98 and 2.98 (girls), and 1.03 and 3.06 (boys); 21-year-olds had cut-offs of 0.93 and 3.05 (girls) and 1.00 and 3.41 (boys). As this method makes many assumptions, and is particularly insensitive at the individual level when the duration of dietary record is short [16], we did not exclude the "inadequate" reporters from the main analysis. Instead, we present the numbers mis-reporting, and we re-ran all analysis with "adequate" reporters only and identified any significant differences from the main analysis. Basal metabolic rate was estimated from equations developed by Schofield [17].

### Other data

Height and weight were measured according to standardised procedures. Overweight status was determined by comparing a subject's body mass index (BMI, kg/m2) to cut-offs established by Cole et al. [18] for children and adolescents. For the 21-year-old subjects, BMI was compared to the WHO adult reference range [19].

### Statistical analysis

The agreement between tertile membership at EYHS I and tertile membership at EYHS II was estimated in three ways, each assessing a specific aspect of tracking behaviour [8].

1) The proportion of stability is the crudest measure and is simply the proportion of subjects who remained in the same tertile at both time points divided by the total number of subjects. If chance alone determined the tertile at time II, one third would be expected to be placed in the same tertile; a value > 33 therefore suggests that subjects do not stay in the same tertile completely by chance.

2) For overall tracking, Cohen's weighted κ was used [20]. This is a measure of the level of agreement between tertile membership at time I and time II, with membership of the same tertile seen as perfect agreement, but with some weighting given to membership of an adjacent tertile and none to an extreme tertile; e.g. movement from low to moderate is weighted more heavily than movement from low to high. Cohen's weighted κ calculates a value usually between zero and one, where one indicates perfect agreement. The interpretation of a tracking coefficient is subjective, but certain scales have been adopted, for example that of Landis and Koch [21], which judges values of 0.01 to 0.20 as indicative of "slight" agreement; 0.21 to 0.40 as "fair"; 0.41 to 0.60 as "moderate"; 0.61 to 0.80 as "substantial" and 0.81 to 1.00 as "almost perfect" agreement.

3) A predictive value for remaining in the highest tertile was also calculated. This is the proportion of subjects in the highest tertile at time I who remained in the highest tertile at time II, divided by those in the highest tertile at time I who had moved to other tertiles at time II. A value > 1 suggests more subjects stayed than moved.

All statistical analysis was performed using SPSS for Windows, v. 17 (Chicago, USA). Cohen's weighted κ is not available in the standard SPSS package, but command syntax is available by searching the SPSS.com "Knowledgebase" [22]. Data for imputation into the syntax was generated from cross-tabulation. For food groups with high levels of non-consumers, occasionally tertiles were of unequal size, and sometimes only two "tertiles" could be created. SPSS assigns all non-consumers automatically to tertile 1, and the upper cut-off is still made as close to the 66^th ^percentile as possible, which may result in tertiles 1, 2 and 3 being of different sizes. For food groups with very high non-consumption levels, the 33^rd ^and 66^th ^percentile of intake might both be zero; all non-consumers are assigned to "tertile" 1 and the remaining to a second "tertile". If the same numbers of "tertiles" are not available at both time points, predictive values and Cohen's κ cannot be calculated. This was an issue for a total of four food groups. Because of the skewed data, median, 25^th ^and 75^th ^percentile intakes are presented for food groups and nutrients. Data on food group intakes is presented for consumers of those food groups only. The proportion of subjects consuming a food group at time I and time II was compared using a non-parametric paired-sample test (McNemar test). To test for the effect of the predictors age and gender on the outcome of stability, a logistic regression was performed, including an age by gender interaction effect. The level of statistical significance was set at p < 0.05.

## Results

### Participants

Subject characteristics are presented in Table 1. Approximately 40% of subjects returned to EYHS II. As previously described in the EYHS dropout analysis, the older group in EYHS I were less likely than the younger group to return, and boys were less likely to return than girls. At time I and time II, approximately 85% of the sample were of normal weight. In EYHS I, 94% of the sample reported an adequate energy intake, and in EYHS II this was 79%; the percentage adequately reporting at both time points is presented in the table. Inadequate reporting here refers to both under- and over-reporting, but over-reporting was rare (data not shown).

**Table 1:** Participant characteristics

		**EYHS I**					**EYHS II**					
				
					**Weight status**				**Weight status**			
	**Sample**	**Age^a^**	**BMI^b^**		**normal**	**obese**	**Age^a^**	**BMI^b^**		**normal**	**obese**	**Adequate reporting^c^**
	**n (%)**	**years**	**kg/m^2^**	**SD**	**%**	**%**	**years**	**kg/m^2^**	**SD**	**%**	**%**	**%**
				
Younger girls	149 (33)	9.5	17.2	2.3	85.8	1.4	15.5	20.9	2.6	89.3	1.3	79.7
Younger boys	124 (27)	9.5	17.2	2.4	85.4	2.4	15.5	20.5	2.9	84.6	0.8	71.3
Older girls	110 (24)	15.5	21.1	2.5	89.9	0.9	21^d^	22.4	3.3	84.4	4.6	70.4
Older boys	69 (15)	15.5	20.7	2.9	89.9	1.4	21^d^	23.3	3.6	78.3	5.8	81.2

^a ^Mean decimal age

^b ^Mean BMI plus standard deviation (SD)

^c ^Reported adequate energy intake at both time points. See Methods section for definition

^d ^Exact decimal age not available

### Food groups and nutrients

The most commonly consumed food groups (Table 2) were bread; milk, fil and yoghurt; meat and meat dishes; vegetables; and pasta, rice and potatoes - these food groups were consumed by more than 66% of both age groups at both time points. Relative to the percentage at time I, from 9 to 15 years the percentage of consumers of cakes and biscuits; sweets and chocolate and "other sweet things" decreased most, while poultry and poultry dishes; chips and crisps, and cheese increased most. From 15 to 21 years, the numbers consuming sweets and chocolate; spreads and oils, and sweetened drinks decreased most, while chips and crisps, and poultry and poultry dishes increased the most. Intakes of energy, selected nutrients and dietary energy density are presented in Table 3.

**Table 2:** Consumers (%) and intakes of food group consumers (g)

	EYHS I				EYHS II				
		
Younger group			Percentiles					Percentiles	
	%	Median	25^th^	75^th^	%		Median	25^th^	75^th^
	
Bread	94.5	86	50	140	87.5	^a^	100	59	165
Milk, fil, yoghurt	97.4	588	364	824	92.3	^a^	552	352	839
Cheese	40.7	35	20	60	51.6	^a^	40	23	80
Vegetables	79.1	58	30	94	70.3	^a^	65	36	120
Fruit	60.4	150	105	253	56.4		200	105	300
Fruit juice	34.8	206	150	382	35.9		250	200	500
Pasta, rice, potatoes	81.7	225	150	310	75.8		210	130	325
Cereals	44.0	41	20	200	46.5		60	35	150
Pizza, pies, pancakes	24.2	205	140	265	18.7		250	170	350
Meat, meat dishes	83.9	150	70	230	82.4		200	95	320
Poultry, poultry dishes	11.7	100	80	120	18.3	^a^	95	60	200
Spreads and oils	88.3	15	9	25	79.5	^a^	11	6	20
Sweets, chocolate	64.8	16	8	34	39.6	^a^	40	20	89
Cakes, biscuits	49.8	51	30	94	28.6	^a^	56	30	100
Other sweet foods	68.9	65	20	175	49.8	^a^	60	15	140
Sweetened drinks	49.1	252	200	501	46.9		400	250	638
Chips, crisps	16.1	99	16	171	22.7		102	48	150
**Older group**			**Percentiles**					**Percentiles**	
	**%**	**Median**	**25^th^**	**75^th^**	**%**		**Median**	**25^th^**	**75^th^**
	
Bread	97.8	120	75	178	89.4	^a^	105	60	170
Milk, fil, yoghurt	94.4	600	412	931	83.2	^a^	380	200	600
Cheese	61.5	40	21	73	64.8		48	25	80
Vegetables	75.4	100	50	170	77.7		105	55	205
Fruit	52.5	190	105	293	53.6		200	109	366
Fruit juice	36.9	344	206	415	29.6		252	200	435
Pasta, rice, potatoes	70.4	225	150	301	66.5		160	125	235
Cereals	43.0	45	20	175	44.7		59	30	200
Pizza, pies, pancakes	17.3	170	118	280	15.1		250	200	350
Meat, meat dishes	82.7	150	74	214	72.6	^a^	180	86	310
Poultry, poultry dishes	8.4	140	80	200	16.2	^a^	100	60	180
Spreads and oils	87.2	15	9	24	65.9	^a^	12	6	20
Sweets, chocolate	78.8	35	12	87	35.2	^a^	65	25	100
Cakes, biscuits	38.0	60	30	106	38.5		60	38	125
Other sweet foods	58.7	51	17	153	48.0		50	14	150
Sweetened drinks	54.7	412	267	669	43.0	^a^	375	250	600
Chips, crisps	27.4	100	33	161	34.1		75	35	150

Intakes are for consumers only

Consumers: percent of subjects consuming food group

^a ^Significantly different from EYHS I (McNemar test), p < 0.05

**Table 3:** Intakes of nutrients

	EYHS I			EYHS II		
	
Younger group		Percentiles			Percentiles	
	Median	25^th^	75^th^	Median	25^th^	75^th^
	
Energy (MJ)	8.4	7.0	9.9	9.6	7.0	12.1
Energy (MJ/kg)^a^	0.26	0.21	0.31	0.16	0.12	0.21
Protein (% E)	15.2	13.1	17.2	16.2	13.3	18.5
Fat (% E)	32.1	28.3	36.4	31.3	26.8	37.2
Saturated fat (% E)	14.6	12.7	16.4	14.1	11.8	17.1
Carbohydrates (% E)	52.0	47.6	56.5	51.0	46.0	56.4
Sucrose (% E)	10.1	6.7	14.0	9.8	6.1	13.7
Fibre (g)	14.0	10.6	17.4	17.2	11.6	23.8
Vitamin C (mg)	73.4	37.3	123	69.8	32.5	148
Folate (μg)	194	156	240	236	169	316
Iron (mg)	8.4	6.6	11.0	11.1	7.3	15.9
Calcium (mg)	1113	838	1560	1198	826	1652
						
Energy density (kJ/g)	6.5	5.6	7.6	6.9	5.9	8.3
**Older group**		**Percentiles**			**Percentiles**	
	**Median**	**25^th^**	**75^th^**	**Median**	**25^th^**	**75^th^**
	
Energy (MJ)	10.2	7.5	12.1	9.3	6.7	12.1
Energy (MJ/kg)^a^	0.17	0.13	0.20	0.14	0.10	0.18
Protein (% E)	14.4	11.9	17.3	14.9	12.8	17.9
Fat (% E)	31.2	26.4	35.2	32.6	26.7	38.4
Saturated fat (% E)	14.5	11.7	16.7	13.2	10.3	16.4
Carbohydrates (% E)	54.4	48.7	58.4	50.4	44.2	56.0
Sucrose (% E)	11.7	7.6	16.0	10.1	5.8	13.4
Fibre (g)	16.9	11.8	22.0	19.0	13.3	24.4
Vitamin C (mg)	87.9	37.5	192	74.2	34.4	134
Folate (μg)	235	177	311	239	180	339
Iron (mg)	10.8	7.9	13.8	10.9	7.4	15.7
Calcium (mg)	1312	935	1777	1041	640	1461
						
Energy density (kJ/g)	6.5	5.6	7.6	6.9	5.9	8.3

% E is a percentage of total energy

^a ^per kg body weight

### Tracking coefficients

The tracking coefficients for food groups, energy, selected nutrients and energy density are presented in Table 4. The highest proportion of stability seen was between childhood and adolescence for poultry and poultry dishes; chips and crisps; pizza, pies and pancakes, although these values are likely to be inflated because of the high proportion of non-consumers at both time points. The highest stability values in the older age group were seen for poultry and poultry dishes; pizza, pies and pancakes group, and cakes and biscuits. Again, the low consumption rates of the first two groups should be taken into consideration. Stability did not generally differ by gender, with the exceptions of fruit (where boys were more likely to be stable than girls); cakes and biscuits in the younger age group (girls were more likely to be stable), and chips and crisps in the older age group (boys were more likely to be stable).

**Table 4:** Tracking values

	**Younger group (EYHS I to EYHS II)**			**Older group (EYHS I to EYHS II)**		
		
	**Stability^a^**	**Cohen's κ^b^**	**Predictive value^c^**	**Stability^a^**	**Cohen's κ^b^**	**Predictive value^c^**
	
*Food groups*						
Bread	0.36	0.06	0.76	0.36	0.06	0.50
Milk, fil, yoghurt	0.49	0.30	1.17	0.35	0.06	0.58
Cheese	0.49	0.16	0.86	0.29	< 0	0.45
Vegetables	0.38	0.11	0.55	0.40	0.17	0.79
Fruit	0.40	0.10	0.76	0.49	0.23	0.82
Fruit juice	0.14	< 0	0.45	0.16	n/a	0.67
Pasta, rice, potatoes	0.37	0.06	0.55	0.44	0.14	0.64
Cereals	0.50	0.15	0.77	0.49	0.11	0.69
Pizza, pies, pancakes	0.66	n/a^d^	0.22	0.77	n/a	0.35
Meat, meat dishes	0.34	< 0	0.47	0.36	0.06	0.65
Poultry, poultry dishes	0.76	n/a	0.33	0.78	n/a	0.15
Spreads, oils	0.34	< 0	0.56	0.29	< 0	0.38
Sweets, chocolate	0.33	0.00	0.43	0.41	0.12	0.79
Cakes, biscuits	0.23	n/a	0.48	0.51	0.05	0.61
Other sweet foods	0.37	0.04	0.49	0.39	0.06	0.62
Sweetened drinks	0.44	0.07	0.63	0.48	0.17	0.72
Chips, crisps	0.67	n/a	0.22	0.34	n/a	0.69
*Nutrients*						
Energy	0.36	0.07	0.60	0.33	0.06	0.58
Protein^e^	0.36	0.05	0.57	0.42	0.16	0.67
Fat^e^	0.37	0.06	0.54	0.35	0.04	0.54
Saturated fat^e^	0.38	0.09	0.69	0.36	0.07	0.62
Carbohydrates^e^	0.36	0.06	0.54	0.35	0.03	0.58
Sucrose^e^	0.39	0.11	0.57	0.38	0.07	0.71
Fibre	0.36	0.09	0.65	0.48	0.26	0.94
Vitamin C	0.38	0.04	0.42	0.42	0.19	0.76
Folic acid	0.35	0.02	0.60	0.37	0.09	0.71
Iron	0.35	0.04	0.48	0.34	0.04	0.58
Calcium	0.43	0.18	0.75	0.39	0.06	0.62
						
Energy density	0.35	0.06	0.65	0.40	0.16	0.58

^a ^Proportion remaining in the same tertile six years later

^b ^Cohen's κ, Cohen's kappa (weighted)

^c ^Predictive value for remaining in the highest tertile

^d ^N/a: not applicable because three groups not available at both time points

^e ^As a percentage of total energy

In the younger subjects, Cohen's κ coefficients of largest magnitude were seen for milk, fil, yoghurt; cheese; cereals, and vegetables. In the older age group, fruit; vegetables; sweetened drinks; pasta, rice and potatoes, and sweets and chocolate showed the most agreement. The predictive value of membership of the highest tertile at time II based on membership at time I was highest for milk, fil and yoghurt; cheese, and cereals (but only milk, fil and yoghurt had a value > 1.0) in the younger group. Fruit; vegetables; sweets and chocolate; sweetened drinks, and cereals had the highest predictive values in the older age group (no value was higher than 0.69). For nutrients, stability was highest for calcium from 9 years to 15 years. Cohen's κ was highest for calcium and sucrose (> 0.11), and the predictive values were highest for calcium and saturated fat. Stability in the older age group was highest for fibre, vitamin C and protein. Cohen's κ was highest for fibre, vitamin C and protein (> 0.16). The predictive values were also highest for fibre, vitamin C, sucrose and folic acid. Energy density, a marker of overall dietary quality, had low stability and slight κ values in both age groups.

In summary, in the younger age group, 14 out of the 17 food groups examined had stability values > 33. The milk, fil, and yoghurt group showed fair tracking, and seven others showed slight tracking. Milk, fil, yoghurt also had a positive predictive value for the highest tertile. All nutrients had stability values > 33, and all showed slight tracking. In the older age group, 14 of the 17 food groups had stability values > 33; fair tracking was seen for fruit intake and slight tracking for a further ten food groups. Again, all nutrients had stability values > 33, fibre showed fair tracking and all others showed slight tracking.

### Exclusion of inadequate energy reporters

Re-analysis of the data with inadequate reporters excluded resulted in some small changes to percent consumers of food groups and intakes (data not shown), but the tracking statistics did not differ substantially. One additional food group in the older subjects showed fair tracking (vegetables), and fibre, which had fair tracking, became slight. In the younger age group, folic acid showed no tracking, instead of slight. Stability coefficients were similar. The positive predictive value for milk, fil, yoghurt in the younger group disappeared (became 0.94), while vegetables in the older group became borderline (1.00).

## Discussion

Despite using a dietary assessment method which has known limitations for measuring at the individual level, and over a relatively long time period, we found evidence of slight to fair tracking for many of the food groups and all of the nutrients studied, indicating some stability of intakes at individual level.

### Interpreting tracking coefficients

Interpreting tracking coefficients is hampered by the many ways in which they can be calculated, and a lack of consensus on how to interpret them [8]. The time period over which they are measured is another important factor. A high tracking coefficient over a short time may be just evidence of the reliability of the method used, whereas more modest coefficients over many years, as we generally found, could be stronger evidence of tracking [23]. In cases of food groups where the level of non-consumers is high - a problem when only one day of intake is available - the stability coefficient can be artificially inflated. Another factor that influences the interpretation is the measurement error of the methods used. A single 24-h recall does not reflect usual dietary intake well at the individual level. However, the expected effect of using a method with a large measurement error is to attenuate statistical associations. Therefore, the finding of tracking is noteworthy. In addition to these factors, we assessed tracking across very different age groups. Childhood, adolescence and young adulthood are three distinct life-stages, and the environmental influences on diet vary considerably at each age [5,24]. Children are restricted by what parents and schools provide, adolescents to a lesser degree as both autonomy and the influence of peers increases, while young adults are usually independent and may have moved away from family and where they grew up. If dietary behaviour really does track, it is not clear whether this is due to the stability of food preferences, or to a lasting effect of, for example, parental influences. This is something that deserves further study.

### Evidence from other studies

The current evidence for tracking behaviour in children and adolescents comes from studies varying in design, subject age and methods used. Studies have ranged over periods of one year to several decades, with longer studies usually including more data points. Some have focused on specific food groups (e.g. soft drinks [25,26], or fruits and vegetables [27,28]), some on single nutrients [29], while others have examined overall dietary patterns [30,31]. Several of these studies have, like ours, been part of a larger cardiovascular disease study (e.g. the Bogalusa Heart Study [32], the Young Hearts Project [33], The Cardiovascular Risk in Young Finns Study [31], the CARDIA study [34]). The majority of published studies are from Europe or the USA, but one (English language) study from China [35] was identified via PubMed.

In the study most similar to ours in terms of subject age and tracking method used, Gallagher and colleagues found "substantial drift of subjects between low, medium and high classes of intakes", or slight and fair tracking of macronutrient and micronutrients over 12/13 years, from adolescence to young adulthood [36]. Like us, they used Cohen's weighted κ, but used the lowest 25^th^, the middle 50^th ^and the upper 25^th ^percentiles as their group cut-offs. Using energy adjusted nutrients, κ values remained lower than 0.25. The Amsterdam Growth and Longitudinal Study has examined many aspects of tracking from ages 13 to 32, approximately. They used general estimating equations as they have many data points and have reported moderate tracking of energy in relation to body mass [37], low to moderate tracking for macro- and micronutrients [38] and fruit and vegetables [27]. In a comparable age group and time period, Lake et al have studied five food groups, based on the English national food model and found significant ranking correlations for three of them [39]. Over a similar length of time as our study (six years), but in much younger children (ages 3 and 4 at baseline), the Framingham Children's study found evidence of tracking, particularly for carbohydrate and fat [40]. Our results are in line with those of these studies, although the diversity makes it hard to compare directly.

### Usefulness of tracking coefficients

Being able to predict future intakes from current intakes is of interest, both for nutritionists concerned about whether certain habits, for example, high sweetened drink consumption in adolescence, will remain high later on in life, or for those who want to know if high intakes achieved at a particular life-stage will have a lasting effect. We calculated a predictive value for remaining in the highest tertile of intake to help to answer questions such as: "what is the likelihood of someone with a high intake of vegetables at time I having a high intake at time II"? Low fruit and vegetable intakes are a target of public health campaigns in many countries, including Sweden, and the results of this study would suggest that high vegetable intakes in childhood do not predict high intakes in adolescence, whereas high fruit intakes are more likely to remain high. High intakes of vegetables in adolescence on the other hand are more likely to remain high in young adulthood. However, only one food group, in the young age group, had a positive value for predicting membership of the highest tertile at follow-up (the milk, fil, yoghurt group).

The tracking of dietary intakes is important to study as it has implications for public health planning and epidemiology. From our results, it is not possible for us to conclude that the diet appears more stable from childhood to adolescence than from adolescence to young adulthood, or vice versa; both age groups showed comparable tracking values. While, statistically, we did see evidence of dietary tracking, it is also clear that many subjects do not remain in the same tertile, and so their behaviour, in both childhood and adolescence, appears potentially modifiable. This is potentially encouraging for health promoters and intervention planners. For epidemiologists, based on our results, in these age groups it would appear necessary to use a different dietary record method or more frequent data collections to be confident of classifying the intakes of these children and adolescents correctly.

### Limitations and strengths of the study

Although this study presents data on two groups of 15-year-olds, one from 1999 and one from 2004, attempts to examine temporal changes in intakes should be avoided. Given the participation and dropout bias it would be inappropriate to make assumptions about changes in the behaviour of 15-year-olds over time, based on this data. To detect temporal effects a more representative sample would be required. For this reason, certain differences in food and nutrient intakes between the two groups (which can be detected on examination of Tables 2 and 3) have not been highlighted or discussed.

Issues specific to the longitudinal study of diet are changes in food availability, food composition and promotion [41]. This could account for some of the changes, such as the large increase in poultry and poultry dishes consumption in the six years, which are promoted as healthier alternatives to red meat. The mis-reporting of dietary intake is a significant challenge to the quality of any dietary study. The simplest way to check for the presence of mis-reporting is to compare reported energy intakes to the estimated energy requirements, assuming weight stability of the subject. The method developed by Goldberg and colleagues [14], and elaborated on by Black [16], establishes cut-offs around this expected energy value, taking into account variability in dietary intake, variability in energy expenditure, and length of dietary record. The proportion of subjects who reported "inadequate" energy intakes in our sample was in line with other studies, but wide ranges (from 10 to 50%) have been reported [42]. Unfortunately, the Goldberg method is not particularly sensitive at the individual level, especially when only one day of dietary data is available. We decided not to exclude inadequate reporters but rather to consider the effect that excluding them may have had on the results. This has been recommended as a minimum standard [43]. We found that excluding them had no substantial effect on the overall picture of tracking. All longitudinal studies are prone to the loss of subjects to follow-up. We have considered the potential influence this dropout might have on our study with a separate dropout analysis. The participation and dropout rates affect how the results of this study could be generalised to the general population. This is certainly the case when it comes to the median intakes, for example. However, we are not aware of any obvious theoretical reason to suspect that the tracking we observed would necessarily be any different in a more diverse population. In addition, data is presented at group level where possible, which is more appropriate for dietary data of this type [44]. The time period between data collections is quite long, and the availability of a third data point could have improved the study. Again, the longer time period would be expected to decrease the likelihood of detecting tracking. The 24-h recall interviewers were not the same in both years, but the training procedure and the trainers were. An updated nutritional database could account for some of the differences in nutrients seen, but this has no effect on intakes of food groups or ranking. Otherwise, the dietary data was processed and handled in the same way at both time points. Another strength of this study is that we have considered the effect of energy mis-reporting on our results. That energy mis-reporting is a huge issue for nutritional epidemiology is gaining recognition, but many studies still fail to address this clearly, and others fail to mention it at all.

## Conclusions

Evidence for slight tracking was observed for most food groups and nutrients, indicating some modest stability of diet at individual level. This was despite the long time period between data collections, and the method of data collection used. However, many subjects do not maintain their ranking, implying that the diets of both children and adolescents are potentially modifiable. This topic warrants further study because of the relevance it has for the development of public health nutrition strategies in these age groups and for the design of future nutritional epidemiological studies.

## Competing interests

The authors declare that they have no competing interests.

## Authors' contributions

EP performed the analysis and drafted the manuscript. JW helped to plan the analysis and draft the manuscript. JK helped to draft the manuscript. MS was responsible for the study, oversaw data collection and helped to draft the manuscript. All authors read and approved the final manuscript.
